# Concealed retropharyngeal haematoma: A rare but life threating complication following a low risk fall

**DOI:** 10.12669/pjms.38.8.7147

**Published:** 2022

**Authors:** Nosheela Rafique, Ehtesham Khan, Paula Connolly, Shankar Lal

**Affiliations:** 1 Dr. Nosheela Rafique, FCPS. Registrar, Anaesthesia Department, Our Lady of Lourdes Hospital Drogheda, Rep of Ireland; 2 Dr. Ehtesham Khan, FCARCSI. Chair of Credential Committee, Council Member CIA, Consultant Anaesthesia, Our Lady of Lourdes Hospital Drogheda, Rep of Ireland; 3 Dr. Paula Connolly, FCARCSI. Consultant Anaesthetist, Our Lady of Lourdes Hospital Drogheda, Rep of Ireland; 4 Dr. Shankar Lal, FCARCSI. Registrar, Anaesthesia Department, Beaumont Hospital Dublin, Rep of Ireland

Retropharyngeal haematoma is a rare condition presenting following a severe neck trauma, retropharyngeal inflammation, rupture of vertebral artery aneurysm or as post-surgical complication. We wish to present a case report of an 85-year-old man who developed concealed progressive neck swelling following a low risk mechanical fall and required urgent airway management due to rapidly developing stridor and loss of consciousness. The patient’s presentation was very atypical, he initially presented to ED with GCS of 15/15 and mild neck swelling with absence of any motor and sensory deficit. There was no evidence of stridor, dyspnoea or hoarseness. CT series for trauma was performed taking spine precautions. The initial CT revealed oesophageal dissection with mild pressure effects to surroundings tissues with no evidence of skeletal injuries and neck haematoma but within no time patients deteriorated and developed stridor along with drop in GCS down to 13/15. Thus the decision to secure the airway in a controlled environment (OT) was made with ENT team standby. After multiple failed nasal fibreoptic intubation attempts trachea was intubated by a anaesthetist consultant using video laryngoscopy, bougie and smaller size endotracheal tube. Patient remained hemodynamically stable throughout procedure with nadir saturation of 94%. The subsequent management of the patient was done in the ICU. The patient died two days after ICU admission; he remained hypotensive despite aggressive resuscitation and inotropic and vasopressor support. The post-mortem was done, and it revealed huge retropharyngeal haematoma, Myocardial Infarction (MI) and critical aortic stenosis.

Retropharyngeal hematoma with impending upper airway obstruction leads to a life-threatening clinical situation due to their proximity to the airway, requiring rapid diagnosis and management.^1^ Common causes of retropharyngeal hematoma includes anticoagulant therapy, iatrogenic injury, infections, foreign body ingestion, vascular lesion and major trauma involving cervical spinal fracture.^2^ Slowly growing neck hematomas can be undiagnosed or misdiagnosed, especially when the index of suspicion is low.

In our case, the initial radiological examination did not reveal any definitive diagnosis in terms of hematoma, CT-angiography with contrast, was done and it revealed most likely bleeding but nothing else. This is a very rare presentation of concealed and expanding cervical hematoma in retropharyngeal space which was confirmed on post-mortem diagnostic investigation.

In agreement with the other author’s findings we strongly recommend early management of the airway in suspected cases of retropharyngeal haematoma. The hematoma usually resolves by itself over a few weeks; however, the one rapidly expanding needs to be evacuated surgically, aspirated trans-orally or can be treated by embolization. Some authors encourage a surgical airway in literature as the preferred method over endotracheal intubation due to difficulties associated with intubation. However, it carries the risk of perforating the hematoma. Retropharyngeal hematoma is a rare and potentially fatal presentation with progressing airway obstruction. Early recognition and securing the airway are the key to managing these cases.

## Authors’ Contribution:

**NR and SL:** Data collection and initial write up. **PC and EK:** Final write up.
